# Using machine learning to detect the differential usage of novel gene isoforms

**DOI:** 10.1186/s12859-022-04576-3

**Published:** 2022-01-18

**Authors:** Xiaopu Zhang, Musa A. Hassan, James G. D. Prendergast

**Affiliations:** The Roslin Institute, University of Edinburgh, Easter Bush Campus, Midlothian, EH25 9RG UK

**Keywords:** Isoform usage, Differential expression, RNA-seq, Machine learning

## Abstract

**Background:**

Differential isoform usage is an important driver of inter-individual phenotypic diversity and is linked to various diseases and traits. However, accurately detecting the differential usage of different gene transcripts between groups can be difficult, in particular in less well annotated genomes where the spectrum of transcript isoforms is largely unknown.

**Results:**

We investigated whether machine learning approaches can detect differential isoform usage based purely on the distribution of reads across a gene region. We illustrate that gradient boosting and elastic net approaches can successfully identify large numbers of genes showing potential differential isoform usage between Europeans and Africans, that are enriched among relevant biological pathways and significantly overlap those identified by previous approaches. We demonstrate that diversity at the 3′ and 5′ ends of genes are primary drivers of these differences between populations.

**Conclusion:**

Machine learning methods can effectively detect differential isoform usage from read fraction data, and can provide novel insights into the biological differences between groups.

**Supplementary Information:**

The online version contains supplementary material available at 10.1186/s12859-022-04576-3.

## Introduction

The number of unique mRNA isoforms encoded by the human genome is estimated to be 3–10 times higher than the number of genes [1, 2]. This transcript diversity enables increased downstream phenotypic complexity through the expansion of the set of proteins encoded by a comparatively small set of genes [3, 4]. Precursor messenger RNA (pre-mRNA) splicing is an important driver of this isoform diversity where the canonical exon, intron and other untranslated regions (such as the 5′ untranslated region, UTR) can be rearranged to generate different transcripts via alternative splicing (AS) [5, 6]. Although AS is often condition- and/or tissue-specific, high-throughput RNA sequencing has revealed that AS occurs in almost all human pre-mRNAs. Further drivers of isoform diversity include the use of alternative transcription start and polyadenylation sites, with these processes often co-occurring to further expand isoform variety [7].

Differential isoform usage between groups has been linked to a wide range of diseases [8–10]. For example, changes in the relative ratios of expressed isoforms of the microtubule associated protein tau (*MAPT*) gene have been linked to tauopathies such as dementia [10]. Likewise, differential isoform usage is a characteristic feature of many cancers. For example, the preferential usage of an isoform of the epidermal growth factor receptor (*EGFR*) gene lacking exon 4 [9]. Consequently, the study of differential isoform usage has the potential to provide important insights into the biological mechanisms underlying diseases and phenotypic differences between groups.

RNA sequencing (RNA-seq) is commonly used to compare the transcriptional profiles of cells and tissues. Although the primary focus of RNA-seq studies is commonly the comparison of absolute expression levels of genes or individual transcripts, it can also be used to study differential isoform usage across populations, tissues or disease states [11–14]. However, these studies can be impeded by a lack of knowledge on the total set of different isoforms produced by a gene, particularly when working with non-model organisms. Although several approaches and tools are currently available for studying differential isoform usage, they often depend on the quality of transcript structure annotation. For example, the GEUVADIS consortium [13] used the ratio of the expression of each annotated transcript to the gene’s total expression level to compare isoform usage between populations. This approach, therefore, relies on the quality of the transcript annotation, with any unannotated transcripts not being tested. The DEXSeq and edgeR software test the relative usage of each individual exon, but again depend on the quality of isoform annotation [15, 16]. To overcome the limitation of poor transcript models, some approaches attempt to derive novel annotation data from the sequencing data itself. For example, DIEGO [11] detects differential isoform usage by first identifying the locations of splice sites using split RNA-seq reads, i.e. reads that map across a splice junction. Cufflinks [12] and Stringtie [14] take this further and attempt to assemble whole transcripts from the RNA-sequencing data to generate a new annotation set that can then be used in downstream differential analyses. However, the generation of high-quality annotations using these approaches has been shown to often require extensive sequencing data to accurately reconstruct all the isoforms of a gene.

The use of machine learning (ML) approaches is becoming increasingly popular in the biological sciences [17, 18]. The scale and complexity of biological data makes it well suited to ML techniques including for the detection of AS. For example, Jaganathan et al. [17] used a deep neural network trained on annotated pre-mRNA sequences to predict splice junctions in other pre-mRNA sequences, showing that many sites of AS were driven by genetic variants linked to autism and intellectual disability. Zhang et al. [18] developed DARTS, which can infer differential isoform usage between samples, by using deep learning trained on specific RNA-seq libraries where RNA binding proteins involved in splicing had been knocked down. A limitation of such ML approaches is that they are also dependent on high quality annotation and/or specific training datasets.

In this study we explored the effectiveness of ML approaches to detect putative differential isoform usage between Europeans and Africans in the absence of high-quality isoform annotation or specific forms of RNA sequencing data. In its simplest form, differential isoform usage is characterized by differences in the relative rate of transcription of different parts of the gene. For example, exon skipping in one group versus another will lead to differences in the proportion of reads mapping to this exon between the groups. By dividing each gene region into windows, we investigate the ability to differentiate groups by the proportion of reads mapping to each window in both simulated and real RNA-seq data.

We hypothesized that due to the relatively small number of samples available in most RNA-seq datasets compared to the number of potential windows in a gene, penalized regression approaches such as elastic net regression may be most appropriate for this kind of problem. As has been shown in the related problem of predicting gene expression levels from genetic variants [19]. However, we also contrast this approach to other ML methods to identify which are best suited to this problem and illustrate how our results compare favourably to previous non-ML methods.

## Methods

### Real RNA-seq dataset and data preprocessing

The real RNA-seq data used in this study was previously described in Lappalainen et al. [13]. Briefly, stranded RNA-seq data was obtained from lymphoblastoid cell lines (LCL), a commonly used biological material generated by in vitro infection of B cells from peripheral blood with the Epstein–Barr virus. The dataset contains 91 Americans of European descent (CEU), 95 Finns (FIN), 94 British (GBR), 93 Toscani (TSI) and 89 Yorubans (YRI). The 373 samples of European origin were combined into one group (EUR), meaning in total there were 462 samples available for downstream analyses. The pre-aligned BAM files for all samples were obtained from the EBI database (https://www.ebi.ac.uk/arrayexpress/files/E-GEUV-1/processed/).

### Simulated RNA-seq dataset

We simulated different sample sizes, reads depths, and the ratio of samples affected and unaffected by AS across 513 multi-exon genes located on chromosome 21 using the R package ASimulatoR [20]. We tested groups of 250, 500, and 1000 individuals with 3 different chromosomal reads counts (100,000, 400,000 and 1,000,000). For each gene, 80%, 70%, and 60% of samples affected by AS were labelled as group 1, while the remaining unaffected samples made up group 2, leading to a total of 27 simulated datasets. We set an equal frequency of the 9 different forms of alternative splicing events supported by ASimulatoR (exon skipping, multiple exon skipping, intron retention, alternative 3′-splice site, alternative 5′-splice site, mutually exclusive exons, alternative first exon, alternative last exon and combinations of each) across the 513 genes (Additional file 3: Figure S2), with the same gene influenced by the same AS event across all 27 datasets. To estimate the false positive rate of differential isoform usage detection, we also simulated the same 3 sample sizes and read counts again but with no genes with an AS event. In these cases, samples were simply randomly labelled as group1 and group2 with the same splits as before. We set the number of windows per region (n_w_ see below) to 3 and flanking length to 1.5 kb across these analyses.

### Windowing approach

For both the simulated and real GEUVADIS RNA-seq data the exact same data preparation and modelling approaches were used. To define the windows to be used as features in the models, we first used Bedtools [21] to merge overlapping exons of each Ensembl gene (GRCh37.87), analogous to the flattening approach adopted by DEXSeq [15]. This reduced each gene to a single model. Introns and up/downstream regions before the first and after the last exon were included as well. Each exon, intron, and flanking region in this model was then divided into an equal number of windows (n_w_), meaning the total number of windows/features for a given gene was (num_exons_ + (num_exons_ − 1) + 2) × n_w_ or more simply (2 * num_exons_ + 1) × n_w_ where num_exons_ is the number of exons in that gene following merging. We set n_w_ to 3, 4, and 5, and flanking region lengths to 1 kb, 1.5 kb, and 2 kb to construct 9 candidate input datasets for each gene when characterizing the importance of these parameters. When evaluating windowing numbers, we utilized all 57,736 genes annotated in Ensembl.

Next, Bedtools [21] was used to count the number of RNA-seq reads from each individual in the RNA-seq datasets that overlapped each window. Where a read overlapped multiple windows, it was added to the count for each. If the total read count across a gene was less than 10 for a sample, this sample was excluded when evaluating the gene. Equation 1 below, was used to calculate the normalised proportion of reads mapping to each window, accounting for the window’s length.

1 $$Normalised\;Proportion = \frac{{win\;reads{/}gene\;reads}}{win\; length }$$

### Model fitting and variable importance

Using the approach above we constructed an individual matrix of normalized read counts for each gene as input data for the machine learning models. Each row was a sample/individual and each column a feature/window. The windows and the population codes (group1/group2 in the simulated datasets and EUR/YRI in the real dataset) were designated as the features and sample labels, respectively.

For each gene, we randomly split all samples into training and testing sets with an 80%:20% ratio. Before fitting the models to the input matrix, for each variable (i.e. window) we subtracted the mean and divided by its standard deviation using the “center” and “scale” methods in the preProcess function of the caret R package. To remove uninformative predictors, variables identified with constant or almost constant value across samples were excluded using the nearZeroVar function in caret with its default settings. Tenfold cross-validation with 5 repeats was used in all downstream model fitting. We compared the performance of four model types using the R package caret: logistic regression, elastic net, random forest, and gradient boosting (xgboost). Logistic regression was treated as the baseline model to investigate the effect of changing the flanking length and n_w_ parameters discussed above. Variable importance was evaluated through specific model-based approaches, as implemented by caret. The corresponding R code, specific details on hyperparameter tuning, and parameters used for model fitting are available in the 00.runCaret.R script available at https://github.com/ZXiaopu/IsoformUsage/tree/main/dataAnalysis.

### Permutations and FDR calculations

To assess the number of gene models expected to show evidence of differential isoform usage between the two populations by chance in the GEUVADIS data, we permuted the population labels in the training set between the samples ten times. We then repeated the model fitting and assessed model performance as with the unpermuted testing data. By permuting the training data population labels in this way, we could assess the number of significant gene models that were returned even when the link between population labels and underlying windowed read proportion data had been broken. This provided an estimate of the proportion of results at different *P* value thresholds that were likely false positives, from which we could derive false discovery rate estimates.

To characterize the potential impact of gene expression levels on the number of false positives, we broke the genes down into three approximately equal sized groups based on their expression levels in the European population. Those with an RPKM ≤ 0.005, RPKM > 0.005 and < 1, and those with an RPKM ≥ 1 (RPKM: reads per kilobase per million mapped reads). The gene expression level information was provided by GEUVADIS and available at https://www.ebi.ac.uk/arrayexpress/files/E-GEUV-1/analysis_results/. We then counted the number of genes in each of these categories in each *P* value bin and repeated the enrichment analysis comparing the numbers in the real versus permuted results.

### Comparison with GEUVADIS consortium and edgeR

We compared this windowing approach to two existing methods. The approach originally adopted by the GEUVADIS consortium [13] and that implemented in the R package edgeR [16]. The GEUVADIS consortium had identified potential differential isoform usage by comparing each transcript’s expression level to the whole expression level of its cognate gene using a Mann–Whitney test. These *P* values were then corrected using the Benjamini–Hochberg FDR method. Where this ratio is different between the two populations, this suggests the relative usage of the particular isoform has changed. To implement this method, we downloaded the transcripts and gene expression level files from https://www.ebi.ac.uk/arrayexpress/files/E-GEUV-1/analysis_results/. The gene annotation was from Gencode v12 and transcript annotations from a combination of both Gencode v12 and FluxCapacitor. After repeating this process, we selected only genes with at least one transcript with a corrected *P* value less than 0.05 for downstream analyses.

edgeR tests for differential isoform usage by comparing the log-fold change of individual exons between populations within a gene. To convert the exon level results into gene-level *P* values, the F test and Simes method implemented in edgeR were used. To generate the input read counts from the same BAM files used for the other methods we used HTSeq [22]. Lowly expressed genes (less than 15 reads across all samples) were removed using edgeR’s filterByExpr function with its default settings. When evaluating the outcome, we corrected *P* values using the Benjamini–Hochberg FDR method and only selected genes with a corrected *P* value less than 0.05 for both the F test and Simes gene level results. All code is available at https://github.com/ZXiaopu/IsoformUsage/tree/main/dataAnalysis.

The number of genes found by each set of approaches was plotted using the SuperExactTest R package [23] which also tests for enrichment of any overlaps between groups.

In the downstream analysis, we only evaluated the 50,408 genes which were tested across all ML methods, the original GEUVADIS approach, and edgeR.

### Pathway enrichment analyses

Pathway enrichment analyses were performed using FUMA [24]. The background list was the 50,408 genes described above. To compare approaches, the unadjusted *P* values from across terms and annotation groups tested by FUMA were first log transformed and then compared using a Pearson’s correlation.

## Results

### Population RNA-seq datasets

To investigate the ability of ML approaches to detect differential isoform usage from windowed read proportions we used the GEUVADIS RNA-seq dataset generated by Lappalainen et al. [13]. This study generated stranded lymphoblastoid cell line RNA-seq data across 373 Europeans and 89 Africans. This dataset is not only one of the largest RNA-seq studies spanning multiple groups, but it has also previously been used to investigate differential isoform usage, providing a benchmark for this study.

### Using machine learning models to detect differential isoform usage in the GEUVADIS dataset

**Fig. 1:**
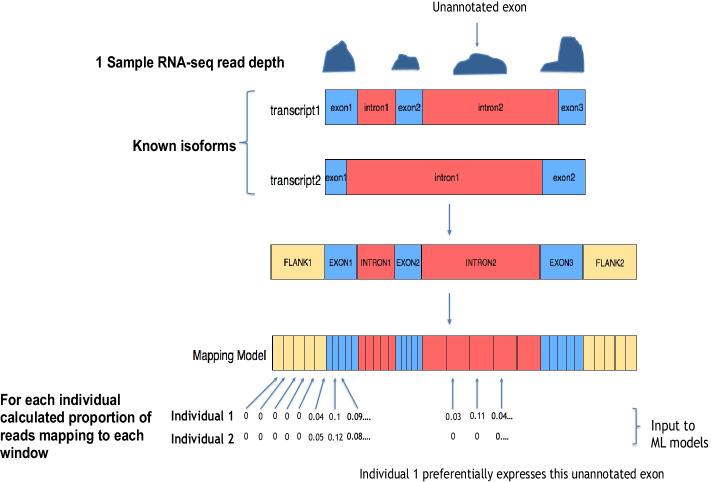
An outline of the windowing approach adopted in this study. For each gene all known transcripts were first merged. Each region (upstream/downstream flanking sequence, introns, and exons) was then divided into a set number of windows. The proportion of reads mapping to each window was then calculated as described in the methods and used as input to the ML approaches

As discussed above, a characteristic of differential isoform usage is a change in the proportions of reads mapping to different areas of a gene. Where an isoform is previously uncharacterised, these changes can occur not only at known exons, but also in intronic or flanking regions. To detect potential differential isoform usage between populations, we first divided each Ensembl gene region (GRCh37.87) into windows and calculated the normalized proportion of reads mapping to each, as illustrated in Fig. 1. By including windows in the upstream and downstream flanking regions as well as introns, we ensured that unannotated isoforms could be captured. All sub-regions were divided into an equal number of windows (n_w_), so that longer regions, such as introns, did not have a disproportionate contribution to the total window number. Consequently, in downstream model fitting, the normalized proportion of reads mapping to each window of a given gene were the set of features, with the population of origin of the individual (EUR or YRI) being the group labels.

**Fig. 2:**
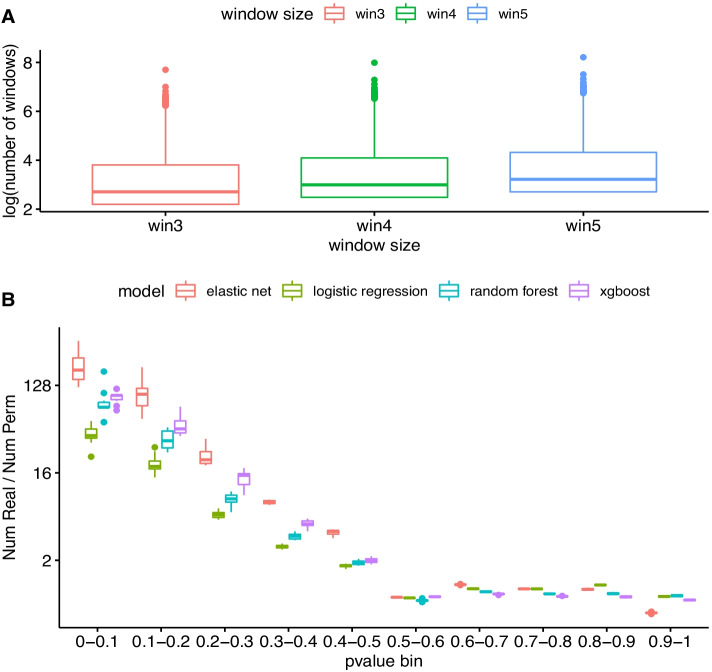
**A** Total window number of genes for different n_w_. **B** To assess the number of significant gene models we would expect to get by chance, we repeated the model fitting ten times but first permuting the population labels in the training set between samples each time. The test dataset was left unchanged. The number of models with *P* values within the given thresholds was then divided by the number observed in each permutation run to see how many more significant results were obtained than expected by chance. As can be seen there is an enrichment specifically of smaller *P* values in the unpermuted data

The n_W_ variable is likely to be important as too many windows will increase the potential for overfitting, and lead to too few reads per window to accurately calculate read proportions. In contrast, too few windows will reduce resolution and the ability to capture novel isoforms. Thus, we examined all the 57,736 genes annotated in Ensembl (GRCh37.87) to identify a reasonable value for n_W_. As shown in Fig. 2A, when n is set to 3, 4 and 5 the average total number of windows per gene was 36, 48 and 60, respectively. However, there is large variation around these values with 5% of genes having more than 135, 180, and 225 windows at each of these thresholds. Using a baseline logistic regression model, we compared the impact of varying n_w_ and the length of the flanking regions on the estimation of model accuracies. In general, a window number of 3 and a flanking region length of 1.5 kb identified the relatively highest number of genes showing differential isoform usage in both the training and testing datasets with less evidence of substantial overfitting across models (Additional file 3: Table S1, Fig. S1). We therefore used these parameters in downstream analyses.

### Using machine learning to identify genes showing differential isoform usage

We used four different modelling approaches to investigate whether ML could effectively detect differential isoform usage between Europeans and Africans from the normalized windowed read proportions. As discussed above, we chose elastic net penalised regression due to the relatively small number of samples relative to the total number of windows for some genes, but also compared the performance of a baseline logistic regression model, random forest, and gradient boosting methods (see Methods).

For each gene, we divided the samples into the same 80% training and 20% testing data and trained models to predict an individual’s population from the distribution of reads across each gene region. If a gene’s model predicts an individual’s population of origin from windowed read proportions better than expected by chance, this suggests the populations display differential isoform usage at the given gene. The number of genes with small *P* values was substantially higher than when the population labels in the training dataset were permuted between the samples prior to model fitting (Fig. 2B), suggesting an enrichment of true positives across all models. However, the greatest enrichment was observed for the gradient boosting model with 8978 genes with an FDR estimated from these permutations of < 0.05. The baseline logistic regression model performed the worst with a comparatively small number of genes with a significant accuracy in the testing data beyond what would be expected by chance.

To further assess the performance of this approach, we estimated potential true positive and false positive rates by simulating reads from genes with and without splicing events using the ASimulatoR R package [20] (see Methods). At a similar chromosomal read count (400,000) and cohort size and split (400 vs 100 individuals) as the real data, the gradient boosting model again performed the best, peaking at an estimated sensitivity of 78% with a specificity of 99.5% (Additional file 3: Figure S2B). Note it is difficult to achieve a sensitivity of 100% in these simulations due to the low expression level of some genes, but these simulations suggest that all approaches can identify the majority of true positives while, importantly, maintaining a low false positive rate.

Although lowly expressed genes are expected to show more noise in their estimated windowed read proportions, with small fluctuations in read counts leading to bigger changes in the estimated values, consistent with the simulations, there is little evidence to suggest this is leading to a large number of false positives in the real data. Few lowly expressed genes were associated with a small *P* value, likely due to the difficulty of detecting consistent differences because of these random fluctuations, as in standard differential gene expression analyses. For example, although 581 of the top third most highly expressed genes had a *P* value less than 0.1 in the unpermuted data in the gradient boosting model, this number was just 17 for the genes in the bottom third of expression levels. These numbers were also still higher than those observed among the permuted data (Additional file 3: Figure S3) suggesting there are few false positives arising purely due to inaccurate estimates of windowed read proportions at lowly expressed genes.

### Comparison to other methods for detecting differential isoform usage

To further assess the performance of these models, we first compared the genes identified by each model to those displaying differential isoform usage when using the method implemented in the original GEUVADIS paper. The GEUVADIS approach relied on transcript annotations and calculated the expression levels of each known transcript relative to the total expression level of its gene and compared these values across populations using a Mann–Whitney test [13].

**Fig. 3:**
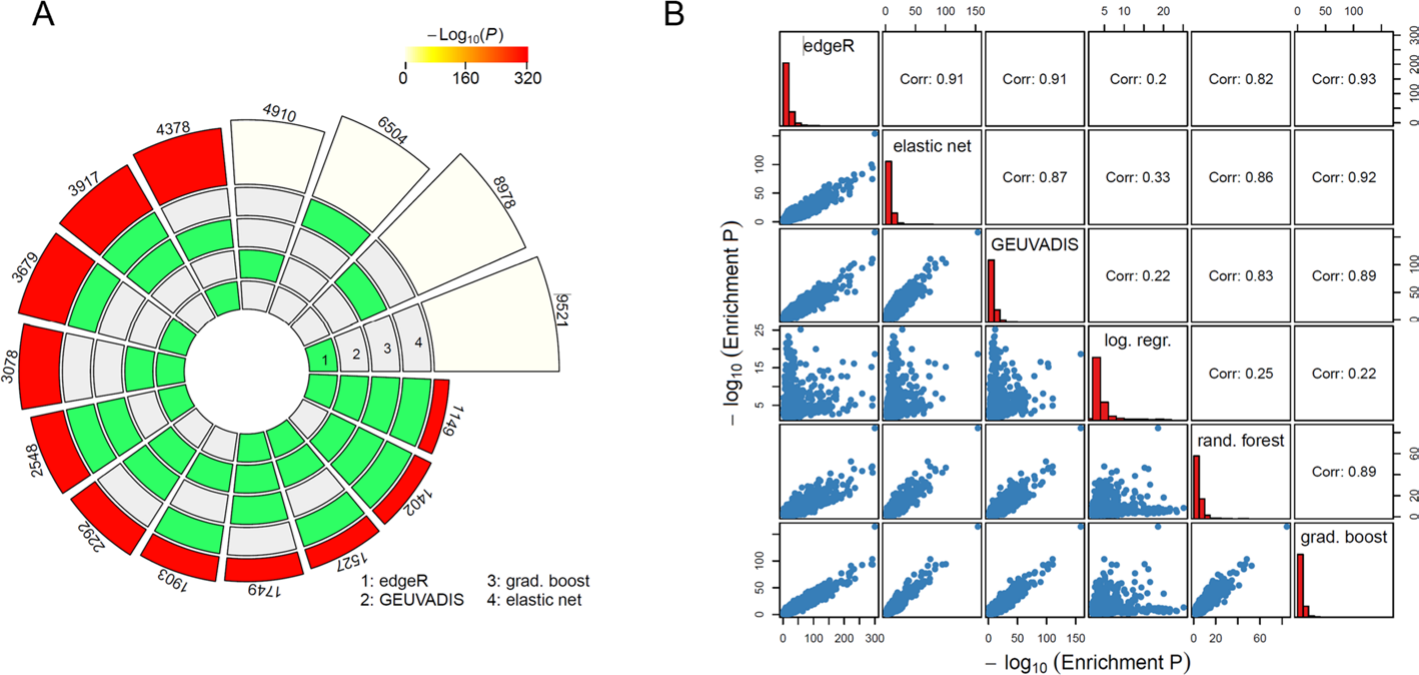
Concordance between methods for identifying differential isoform usage. **A** The number of genes identified by or across approaches. The outer numbers represent the number of genes in each set indicated by the green shading. The red outer shading indicates that set shows greater overlap among the indicated approaches than expected by chance as calculated by the SuperExactTest R package. **B** The concordance of gene enrichment results from FUMA. The log transformed enrichment unadjusted *P* values for tested terms were correlated between approaches. The histogram indicates the distribution of the corresponding log transformed *P* values

The leading ML approaches by FDR identified 25% (elastic net), and 70% (gradient boosting) more genes as potentially linked to differential isoform usage than the GEUVADIS approach (Fig. 3A, Additional file 1: Supplementary Data 1). This is in large part because for many genes there is only one annotated transcript, which limits the number of genes that can be identified as exhibiting differential isoform usage by the GEUVADIS approach. In contrast the ML windowing approach, which relies on read density within a window, can still detect potential differential isoform usage between populations at these genes despite their lack of multiple annotated isoforms. In total 33% and 26% of the genes identified as displaying potential differential isoform usage using the gradient boosting, and elastic net models only had one annotated isoform and could not therefore be detected using the original GEUVADIS approach. In total 57% of the genes identified as showing potential differential isoform using the GEUVADIS approach were detected by either the elastic net or gradient boosting approaches (Fig. 3A).

We further compared the results from our methods and edgeR, which compares the log fold-change of exons across a gene to see where they are inconsistent, suggestive of differential isoform usage [16]. In contrast to the GEUVADIS approach this method is not restricted to analysing genes with multiple known isoforms, though does rely on known exon annotations. 9137 genes were identified as showing potential differential isoform usage with this counts-based method. Highly significant overlaps were observed between the genes identified by all approaches (Additional file 3: Table S2), with 66% of genes (3976) identified using the elastic net approach also identified either by the GEUVADIS or edgeR approaches, with 25% identified by both (1527 genes, 12.8-fold more overlap between the approaches than expected by chance). The corresponding numbers for the gradient boosting model were 58 and 21% respectively. 38% and 45% of the genes predicted to show differential isoform usage by edgeR were also detected by the elastic net and gradient boosting approaches respectively, both higher than the 32% detected by the GEUVADIS approach. Consequently, although all approaches show incomplete overlap due to their different methodologies, each shows a considerably higher overlap than expected by chance, and the elastic net and xgboost models showed better overlap with the edgeR results than the original approach adopted by GEUVADIS.

**Fig. 4:**
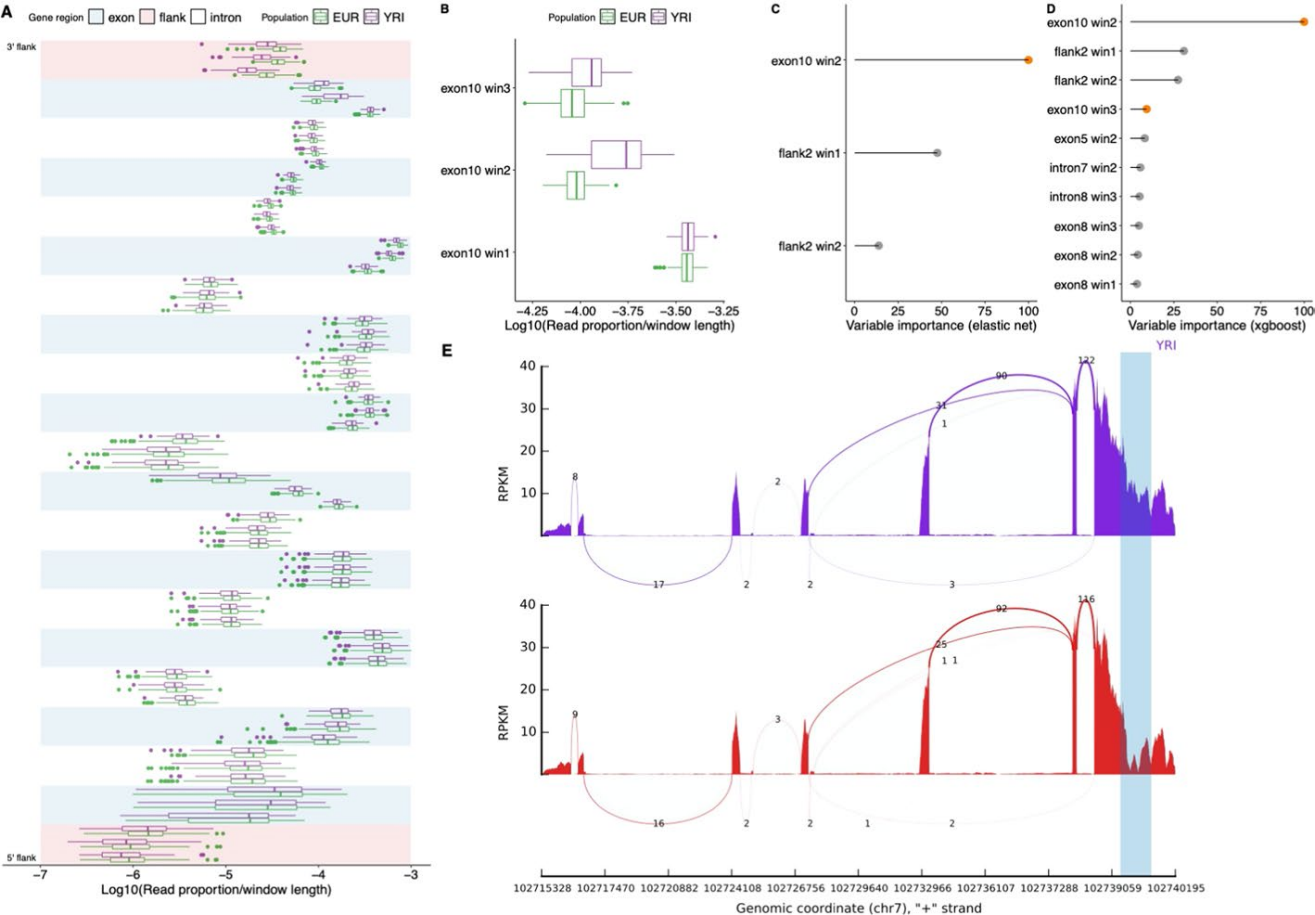
Evidence for differential isoform usage at the *ARMC10* gene. **A** The read proportions by window and population. Exons (in blue) and introns (white) are shown in their genomic order from bottom to top. For each window, read proportions were first divided by the window’s length to account for region size before being log transformed to enable their comparison. **B** The second window (win2) of exon 10 shows one of the largest differences in the proportion of reads mapping to it between the European and Yoruban populations. **C** + **D** The relative importance of the top ranked windows in the elastic net and xgboost models. The windows of exon 10 are indicated in orange. **E** Sashimi plots of two individuals (a Yoruban in purple and European in red) confirming the different read profiles in the final exon. Highlighted region is the exon 10 window 2 region of ARMC10. The plot is generated by rmat2sashimiplot

*ARMC10* is an example of a gene that had an FDR < 0.05 in all four ML models and GEUVADIS but not edgeR. Closer examination of the windowed read proportions across this gene identified the final exon (exon 10) to be particularly informative at discriminating European and African individuals (Fig. 4A–D), with the second window of exon10 ranked top when identifying the most important window to classify the two groups. Differences in the proportion of reads mapping to this final exon was confirmed by visualizing the read depth profiles across this exon in rmat2sashimiplot (Fig. 4E). These results are consistent with the potential preferential use of differential transcriptional end sites (TES) between populations at this gene. In agreement with this, a transcript displaying an earlier TES, ENST00000323735, was the only transcript of *ARMC10* showing significantly different expression level between the two groups (*P* = 0.003) when using the GEUVADIS approach.

In contrast the *PSPH* gene was estimated to display differential isoform usage across all four machine learning models and edgeR but not GEUVADIS. Exon 5 showed the largest estimated importance in our analysis (Additional file 3: Fig S4). which corresponded to three overlapping exons (exons 13, 14, and 15) in the edgeR analysis, where they were also the 3 most significantly differentially expressed exons (exon13 *P*: 1.91e^−116^, exon14 *P*: 2.86e^−102^, exon15 *P*: 2.14e^−43^).

Many genes only identified by our method but not edgeR can be explained by the reads distributed in introns and flanking regions that were considered in our method but not edgeR. For example, among the genes also detected by edgeR the most important window of 60% (2543 out of 4269) and 46% (1675 out of 3604) of genes identified by the xgboost and elastic net approaches were exonic. In contrast among the genes detected by these models but not edgeR these numbers were 34% (1343 out of 3969) and 33% (803 out of 2436) respectively. Consequently, among the genes not also detected by edgeR the most important window is significantly more likely to be intronic or in a flanking region (Fisher’s exact test *P* < 2.2 × 10^–16^), providing a potential explanation as to why many were not also detected by edgeR, that focuses exclusively on known exons.

### Pathway enrichment among genes showing differential isoform usage

A common downstream analysis in gene expression studies is the characterisation of biological pathways enriched among genes identified as showing differences between the groups. Observation of relevant pathways can add support to the validity of the results. We consequently investigated the consistency of functional enrichment among the genes identified by each approach using FUMA [24], and the pathways showing differences between the populations. All FUMA results are provided in Additional file 2: Supplementary Data 2. As shown in Fig. 3B there was a very high correlation between the enrichment results from all of the methods, with the exception of those when using logistic regression. The strongest correlation was between the edgeR and gradient boosting results with a correlation between the log transformed term enrichment *P* values from these approaches of 0.93. Consequently, the enrichment results are highly consistent between methods. 

Enriched terms identified included those likely associated with the different latitudes of the two populations. For example, genes downregulated in fibroblasts in response to UVB irradiation were enriched among all models except the logistic regression model (Additional file 1: Supplementary Data 1). This potentially reflects differential isoform usage linked to the differences in UVB amounts between the two continents. Likewise, chronotype, which is linked to latitude and day lengths was enriched among both the elastic net and gradient boosting results. Genes involved in breast cancer, which have been reported to have a higher incidence in Europeans than Africans, were also enriched among the results of all models [25].

### The relative importance of different gene regions

Elastic net and gradient boosting identified 6504 and 8978 genes displaying potential differential isoform usage at an FDR of 0.05, respectively. Of these a total of 3917 genes (60% and 44% respectively) were detected by both approaches, a significantly higher overlap than expected by chance (*P* < 2.2e^−16^, Fishers Exact test).

To better understand the patterns of differential isoform usage across genes we identified which gene regions generally exhibited the highest relative importance across these two model types. This provides an indication of which regions are more likely to show larger differences between their relative read counts between the two populations, and therefore the location of putative isoform differences.

**Fig. 5:**
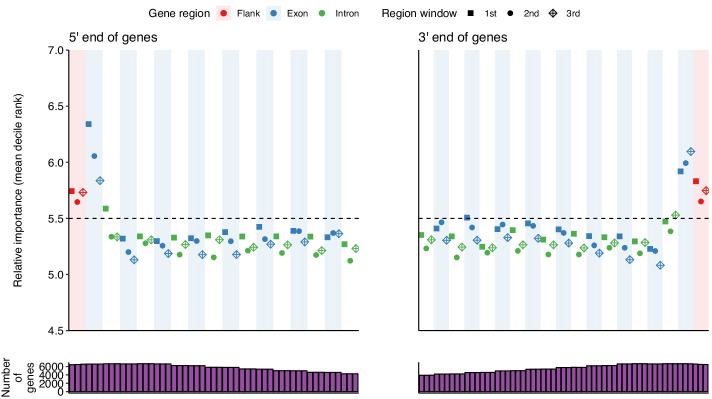
The relative importance of each gene region across multi-exonic genes in gradient boosting models. Relative importance metrics for each gene were converted to deciles, with higher deciles corresponding to higher relative importances within a gene. The mean of each of these for each window was then calculated. Only the first and last eight exons in each gene are shown, along with their corresponding neighbouring introns (green) and flanking regions (red). The dashed lines correspond to the overall means

As shown in Fig. 5, the regions with the largest relative importance were the flanking regions and starts and ends of genes. This suggests primary drivers of isoform differences are likely alternative start and termination sites, consistent with the conclusion from Reyes and Huber [15]. Within introns, the first and last windows showed higher importance than the internal window, consistent with the importance of splice sites. Interestingly, for exons, the last windows generally showed the least importance. Besides the first and last exon, no obvious difference between the importance of the exons and introns of significant genes detected by xgboost (Fig. 5) and elastic net (Additional file 3: Fig. S5) was identified. Within exons or introns there was a low but significant correlation between window length and importance rank (Pearson’s correlation between window length and decile rank among exons was 0.18 and 0.14 in xgboost and elastic net respectively. Corresponding values for introns were 0.13 and 0.09. All *P* values < 2.2e^−16^).

To more specifically investigate any role of alternative splicing, for each gene we identified windows containing a known splice site. These windows were more likely to show a higher variable importance than those that did not overlap a known splice site (*P* value < 2.2e^−16^ for both models, t test). Consequently, despite the importance of alternative transcriptional start and end sites, it is also possible to quantify the potential importance of splice site to driving differences between these populations.

## Discussion

In this study we aimed to investigate whether ML approaches may have utility in detecting differential isoform usage based purely on the distribution of reads across a gene region, even when the precise isoforms are unknown. We illustrated that in the GEUVADIS dataset, gradient boosting can not only identity substantially more genes than expected by chance, but those identified are enriched in relevant biological pathways, consistent with the findings not just being noise.

A major concern for using ML for this type of study is the dimensions of most RNA-seq datasets, in that the number of samples is usually at most measured in the hundreds, substantially less than what would be ideal for employing ML approaches. However, we show that these approaches are picking up many of the genes detected by edgeR and in the original GEUVADIS analysis in a test dataset separate to the training data, and more than expected by chance. Likely due to the dimensions of the data in the GEUVADIS dataset, gradient boosting which is reasonably good at controlling overfitting, showed perhaps the best performance across analyses. Penalized regression was also an effective ML approach in the real GEUVADIS data, consistent with its effectiveness in gene expression imputation analyses where the same restrictions are also present, However, in the simulated dataset elastic net displayed a lower true positive rate though maintained a false positive rate near zero. It is possible any advantages of the elastic net approach are not fully captured using simulated data, for example at longer genes across the genome, or that the false positive rate of this method is higher amongst the real data than predicted. Together these results suggest that ML approaches are indeed likely to have utility in detecting differential isoform usage but the optimal choice for a model may depend on the target dataset and species.

Previous studies have highlighted how RNA-seq can have various biases that can affect the distribution of reads across the gene. For example, read depth is known to be correlated to GC content. However, as the GC content and other sequence features of a given window will be largely the same across groups/populations any such biases should be the same for both, minimizing their impacts.

However, there are a number of caveats to this study. Many of the limitations of standard differential expression analyses will carry over to these analyses in terms of their susceptibility to batch effects etc., but these problems may become amplified when using certain ML approaches. ML approaches are effective at finding differences between groups, though these may not always be biological, and consequently they need to be run with caution. Likewise, although such approaches have the potential to detect differential isoform usage even when the precise isoform is unknown, this likely comes at the potential for increasing the false positive rate. For example, an unknown gene residing in the intron of the gene being studied, will lead to a potential false positive if its expression level is different between the groups being compared. Other current methods based on transcript assembly are also partly susceptible to such issues, but these limitations could be mitigated by using this ML approach as a preliminary screen of genes potentially showing differential isoform usage. Further analysis of paired end and split reads at the identified regions could then be used to confirm the signal is from the gene in question. Further to this, these approaches do not define the actual isoforms showing differential isoform usage and further analyses would be required to characterise these. However, constructing full length isoforms and accurately measuring their expression levels from short-read sequencing data alone is difficult, and often just knowing the gene showing differences is sufficient for many studies to complement the study of differential gene expression. This is the principal that underlies software such as DEXseq [15] that focuses on the differential usage of individual exons. Although in our study we only evaluated differential isoform usage between two groups, it would, in theory, be possible to extend these approaches to comparisons between larger numbers of groups (for example more populations) using multi-class ML models.

Although variable importance metrics need to sometimes be interpreted with caution [26], the data presented here doesn’t suffer from some of the most serious confounders, such as input data of different types. Overall we saw a consistent pattern of the terminal windows being among the most important, supporting the idea that most differential isoform usage is mediated by the use of alternative 5′ and 3′ ends of transcripts. However, we also identified that windows spanning splice sites are also likely to be ranked higher in terms of their importance. Consequently, the results are consistent with underlying biology.

It is notable that whereas there was a significant overlap between the genes identified as showing differential isoform usage using the gradient boosting and elastic net methods, on average half of the genes were specific to each method in each case. This may not be surprising given the very different assumptions and underlying approaches adopted by these ML techniques. It should also be noted that there was an incomplete overlap observed between the results from the previously published edgeR and GEUVADIS approaches. Only 32% of the genes predicted to show differential isoform usage by edgeR were also detected using the GEUVADIS approach. In contrast, 38 and 45% of the genes predicted to show differential isoform usage by edgeR were detected by the elastic net and gradient boosting approaches respectively. There is also often a similar incomplete overlap among the genes identified using different differential expression methods [27], but does mean that the list of genes returned is to some extent dependent on the model and approach used. Integrating different approaches, each with complementary advantages, may prove to be the best strategy for identifying high-confidence genes showing differential isoform usage. Despite this, there were very strong correlations observed among the gene pathways identified across approaches. Reassuringly a number of relevant pathways were consistently identified, including those linked to latitude (UV response and chronotype) and disease (breast cancer) [25].

In summary we demonstrate the potential utility of ML approaches for detecting differential isoform usage. With the ever-decreasing cost of sequencing such approaches will likely become ever more useful in the biological investigation of large RNA-seq datasets.

## Supplementary Information

**Additional file 1**. Genes predicted to display differential isoform usage by each approach.

**Additional file 2**. FUMA pathway enrichment results for each method.

**Additional file 3**. Figures S1 to S5 and Tables S1 to S2.

## Data Availability

All the code used to prepare the data and fit the models is available at https://github.com/ZXiaopu/IsoformUsage. The dataset analyses during the current study are published in ArrayExpress repository (accession: E-GEUV-1), https://www.ebi.ac.uk/arrayexpress/files/E-GEUV-1/processed/.
