# Supplementary material for: Using machine learning to detect the differential usage of novel gene isoforms
Source: BMC Bioinformatics. 2022 Jan 18;23:45. doi: 10.1186/s12859-022-04576-3 (PMC8764765; doi:10.1186/s12859-022-04576-3)
Supplement: Supplementary file 3 — Additional file 3. Figures S1 to S5 and Tables S1 to S2. [file 12859_2022_4576_MOESM3_ESM.docx]

**Supplemental Material**

**Using machine learning to detect the differential usage of novel gene isoforms**

This file includes:

Supplementary Table 1-2

Supplementary Fig 1-5

**Supplementary Table 1**. The numbers of genes with a significant predictive accuracy in the testing sets (top) and training sets (bottom) at various window numbers and flanking sizes.

| n_w_ | 1kb flanking | 1.5kb flanking | 2kb flanking |
| --- | --- | --- | --- |
| 3 | 186 | 210 | 203 |
| 4 | 141 | 147 | 163 |
| 5 | 128 | 130 | 146 |

| n_w_ | 1kb flanking | 1.5kb flanking | 2kb flanking |
| --- | --- | --- | --- |
| 3 | 14455 | 14742 | 14981 |
| 4 | 16648 | 16999 | 17159 |
| 5 | 18432 | 18883 | 19021 |

**Supplementary Table 2**. The observed number of genes identified within and across approaches. The expected overlaps and P values were calculated using the SuperExactTest R package.

| **Intersections** | **Degree** | **Observed Overlap** | **Expected Overlap** | **FE** | **P.value** |
| --- | --- | --- | --- | --- | --- |
| elastic net | 1 | 6504 | NA | NA | NA |
| grad. boost | 1 | 8978 | NA | NA | NA |
| grad. boost & elastic net | 2 | 3917 | 1158.41 | 3.38 | ~0 |
| GEUVADIS | 1 | 4910 | NA | NA | NA |
| GEUVADIS & elastic net | 2 | 1903 | 633.52 | 3.00 | ~0 |
| GEUVADIS & grad. boost | 2 | 2292 | 874.50 | 2.62 | ~0 |
| GEUVADIS & grad. boost & elastic net | 3 | 1402 | 112.83 | 12.43 | ~0 |
| edgeR | 1 | 9521 | NA | NA | NA |
| edgeR & elastic net | 2 | 3679 | 1228.47 | 2.99 | ~0 |
| edgeR & grad. boost | 2 | 4378 | 1695.75 | 2.58 | ~0 |
| edgeR & grad. boost & elastic net | 3 | 2548 | 218.80 | 11.65 | ~0 |
| edgeR & GEUVADIS | 2 | 3078 | 927.39 | 3.32 | ~0 |
| edgeR & GEUVADIS & elastic net | 3 | 1527 | 119.66 | 12.76 | ~0 |
| edgeR & GEUVADIS & grad. boost | 3 | 1749 | 165.18 | 10.59 | ~0 |
| edgeR & GEUVADIS & grad. boost & elastic net | 4 | 1149 | 21.31 | 53.91 | ~0 |

**Supplementary Fig 1.** Comparison of the training and testing accuracies across parameters. Dots are coloured from light to dark to represent the difference between the training and testing accuracies.

**Supplementary Fig 2. ROC curve.** A). 250 samples, B). 500 samples, and C). 1000 samples.

**A.**

**B.**

**C.**

**Supplementary Fig 3. False positives estimation.** False positive rates were evaluated when permuted the sample labels in training sets and the unpermuted training set in elastic net (A) and gradient boosting models (B). When the RPKM is lower than 0.005, the expression level is labelled low while the RPKM is higher than 1, it is highly expressed. Genes with the RPKM from 0.005 to one were regarded as middle expressed.

**Supplementary Fig 4. Evidence for differential isoform usage at the *PSPH* gene.**(A) The read proportions by window and population. Exons (in blue) and introns (white) are shown in their genomic order from bottom to top. For each window, read proportions were first divided by the window’s length to account for region size before being log transformed to enable their comparison. (B) The second and third window (win2, win3) of exon 5 shows two of the largest differences in the proportion of reads mapping to it between the European and Yoruban populations. (C+D) The relative importance of the top ranked windows in the elastic net and xgboost models. The windows of exon 5 are indicated in orange.  (E) Sashimi plots of two individuals (a Yoruban in purple and European in red) confirming the different read profiles in the fifth exon. Highlighted region is the exon 5 window 2 region of PSPH. The plot is generated by rmat2sashimiplot.


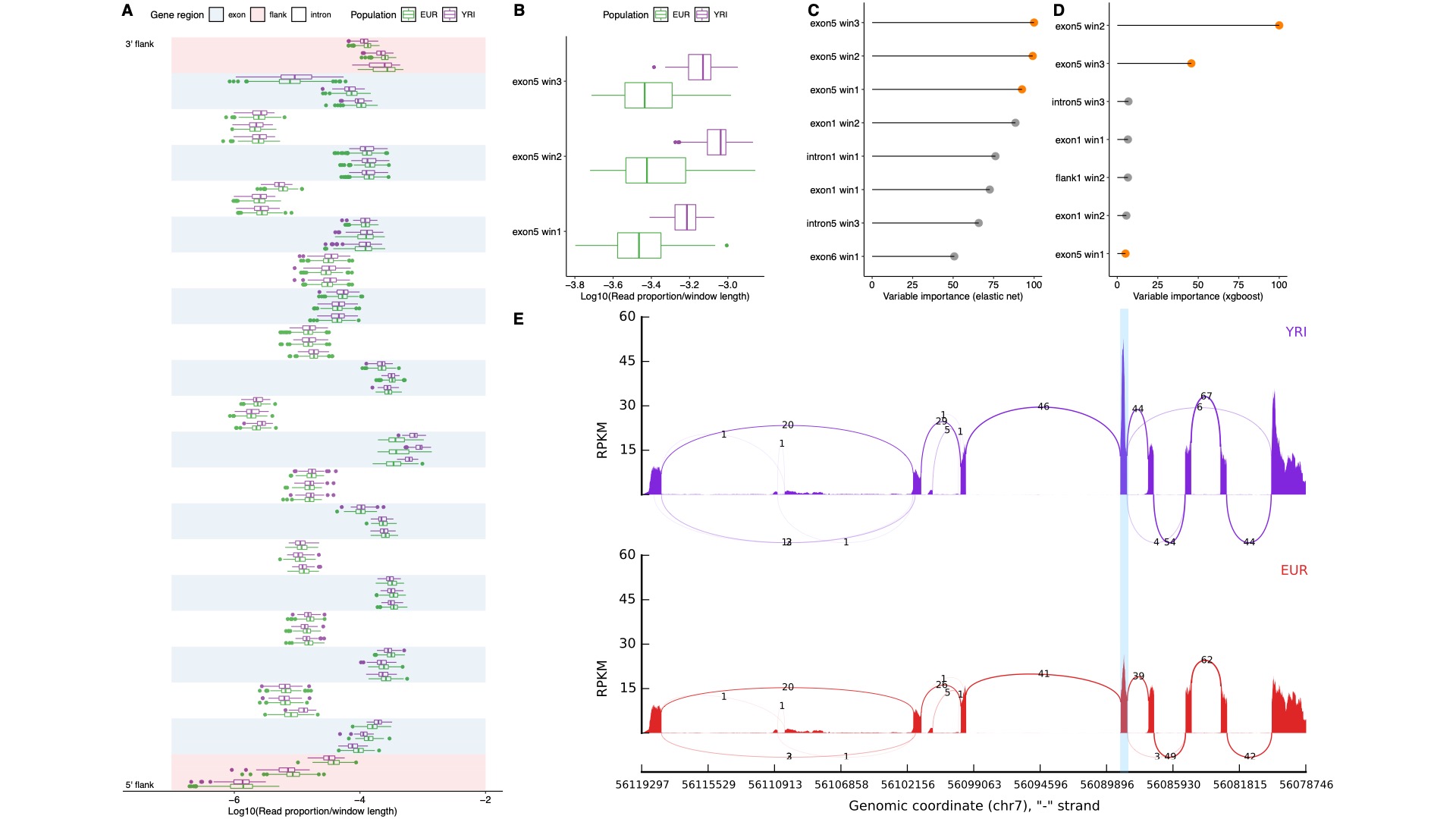


**Supplementary Fig 5. The relative importance of each gene region across multi-exonic genes in elatisc net models.**Relative importance metrics for each gene were converted to deciles, with higher deciles corresponding to higher relative importances. The mean of each of these for each window was then calculated. Only the first and last eight exons in each gene are shown, along with their corresponding neighbouring introns (green) and flanking regions (red).
